# Evaluation of optimal single-photon emission computed tomography reference value and three-dimensional mandibular growth pattern in 54 Chinese unilateral condylar hyperplasia patients

**DOI:** 10.1186/s13005-023-00365-2

**Published:** 2023-05-19

**Authors:** Ningjuan Ouyang, Chenglong Zhang, Feng Xu, Tiantian Chen, Guofang Shen, Jiawen Si, Hongbo Yu

**Affiliations:** 1 Department of Orthodontics, Shanghai Ninth People’s Hospital, School of Medicine, College of Stomatology, National Center for Stomatology, Shanghai Key Laboratory of Stomatology, Shanghai Jiao Tong University, Shanghai Jiao Tong University, National Clinical Research Center for Oral Diseases, Shanghai, 200011 China; 2 Department of Oral & Cranio-maxillofacial Surgery, Shanghai Ninth People’s Hospital, College of Stomatology, National Center for Stomatology, Shanghai Key Laboratory of Stomatology, Shanghai Jiao Tong University School of Medicine, Shanghai Jiao Tong University, National Clinical Research Center for Oral Diseases, Shanghai, 200011 China; 3 Department of Nuclear Medicine, Shanghai Ninth People’s Hospital, Shanghai Jiao Tong University School of Medicine, Shanghai, 200011 China; 4 Department of Oral Implantology, Shanghai Xuhui District Dental Center, Shanghai, 200031 China

**Keywords:** Single-Photon Emission Computed Tomography, Unilateral condylar hyperplasia, Condyle, Mandibular growth activity

## Abstract

**Background:**

The research aimed to evaluate the optimal Single-Photon Emission Computed Tomography (SPECT) cut-off value in differentiating condylar growth activeness, to observe 3-dimensional (3D) mandibular growth pattern, and to explore the potential correlation between 3D measurement parameters and SPECT uptake ratios in Chinese unilateral condylar hyperplasia (UCH) patients.

**Methods:**

Data of fifty-four Chinese UCH patients were analyzed retrospectively. All patients underwent SPECT within 1 month before or after the first CT examination (CT1); and received a second CT examination at least 12 months later (CT2). Data from CT scans were analyzed by comparing bilateral differences between CT1 and CT2. The sensitivity and specificity of SPECT were calculated by the receiver operating characteristic (ROC) curve. Pearson’s correlation analysis was performed to investigate whether the mandibular growth was correlated with SPECT value.

**Results:**

SPECT had a sensitivity of 68.00% and a specificity of 72.41%, with an area under the ROC curve being 0.709. The optimal SPECT cut-off value for evaluating condylar activity has been determined to be 13%. In patients with an active growing condyle, there was a significant increase in Co-Gn and Co-Go, but not in Go-Gn, Go-MF, or MF-Gn. Pearson’s correlation analysis revealed no correlation between 3D measurement parameters and differences in relative condylar uptake ratios.

**Conclusion:**

SPECT showed good diagnostic performance in UCH with the cut-off value of 13%. For those with an active growing condyle, the mandible grows diagonally and vertically, while the relative condylar uptake ratio was not directly related to mandibular growth.

## Background

Unilateral condylar hyperplasia (UCH) is characterized by unilaterally excessive condyle growth, resulting in an inconsistency in the size, shape, and position of the mandible, eventually leading to dental abnormalities, asymmetry deformities, and temporomandibular joint (TMJ) disorders [1, 2]. Based on Obwegeser [3], Wolford et al. defined it as condylar hyperplasia type 1B with an accelerated and prolonged growth aberration of the ‘normal’ mandibular condylar growth pattern, inducing a predominantly horizontal growth vector, resulting in prognathism unilaterally [4]. Even though the etiology remains unclear, mandibular growth of UCH usually ceases at a certain point after a progressive unbalanced growth [5]. Consequently, treatment for UCH depends on the growth activity of the condyle. Thus, it is of utmost importance to choose an appropriate diagnostic method to accurately evaluate the condylar growth potential to determine the appropriate time and method for treatment.

A rapid diagnostic tool known as Single-Photon Emission Computed Tomography (SPECT) has been developed in recent decades to evaluate condylar growth potential, which allows for the acquisition of precise ROIs (Region of Interests) based on anatomical contours and will thus provide higher accuracy in ROI definition [6]. Though the uptake of the radiopharmaceutical in the choice of ROI could be influenced by the size, excellent intra- and inter-observer agreement of SPECT scans was reported when drawing an ROI in the condyles without using a CT scan [7]. Nevertheless, there is no standardized threshold for SPECT relative uptake ratios in assessing condylar growth activity, as different studies exhibited a variation of 5-12% in uptake ratios between normal condyles, with a variation of 10% being mostly reported [8–11]. While some researchers recommended a lower criterion [12] and others recommended a greater one [13], we assume the discrepancy in the diagnostic threshold might be due to different sample sizes, diverse assessment methods, or possible racial differences. Notably, the SPECT criterion for UCH in the Chinese population has yet to be established, and the mandibular growth pattern of Chinese UCH patients with various condylar growing conditions has not been investigated. According to our clinical experience, it is also not uncommon for condyles to remain inactive whereas the uptake ratio is 11% or 12%, and different growing conditions might lead to diverse growing patterns.

Considering the prediction threshold for mandibular growth activity and the growing tendency of UCH patients in the Chinese population has not yet been studied profoundly, this study aims to evaluate the SPECT value to differentiate the condylar growth activeness, to observe the 3-dimensional (3D) mandibular growth pattern and to explore the potential correlation between 3D measurement parameters and SPECT uptake ratios in Chinese UCH patients.

## Methods

### Study design

This retrospective study was conducted at Shanghai Ninth People’s Hospital, Shanghai Jiao Tong University School of Medicine from May 2015 to March 2018. The study was designed under the Declaration of Helsinki and the protocol was approved by the Institutional Ethical Review Board of Shanghai Ninth People’s Hospital (SHDC12013103). Informed consent was obtained from all patients or guardians (for patients who were under 18 but above 16).

Data of fifty-four patients (28 male and 26 female) diagnosed with UCH were analyzed retrospectively in this study, with an average age of 19.87 ± 2.76 years old. The cases had been evaluated clinically and radiologically by experienced maxillofacial surgeons. The diagnosis was made by at least two experts through a combination of the patient’s history and serial assessment of the clinical and radiological findings according to Wolford’s criterion. All of them underwent two visits at an interval of more than one year and an average of 14.43 ± 1.92 months, with complete baseline and follow-up documentation including dental casts, clinical and 3D pictures, X rays and CT scans.

Patients underwent SPECT within 1 month before or after the first CT examination (CT1); and received a second CT examination at least 12 months after the first CT examination (CT2). Patients were excluded if they had a condylar tumor, a history of craniofacial trauma, acquired facial deformity, osteoarthritic diseases or congenital syndromes which may affect the growth and morphology of the mandible and condyle.

The reference criterion was defined according to Chan et al. [14] that if any of the clinical or radiological examinations revealed any changes during the one-year interval, growth was considered active; otherwise, the subject was considered inactive for growth. Thus, patients were classified into three populations based on growth status only or combined with relative condylar uptake ratios. Briefly, those with active condyles were defined as population A, while the subjects with inactive condyles were defined as population I. Patients in population A whose difference in relative condylar uptake ratio was greater than 23%, were defined as population S.

### SPECT acquisition and interpretation

The acquisition method of SPECT was similar to previous description [8]. Briefly, ^99m^Tc-labeled methylene diphosphonate (99mTc-MDP) 555 to 851MBq (14.8MBq/Kg) was injected intravenously four hours before imaging. SPECT was conducted with a SPECT scanner (GE Healthcare, USA) supplied with a low-energy high resolution collimator (LEHR). 140 keV and a 20% symmetrical window were set as the photopeak, and a circular orbit was used for obtaining emission data in the supine position with a matrix of 128 × 128. Each head circulated 180° with 30 stops, with each stop setting at 6°. Coronal, sagittal, and trans-axial tomograms were reconstructed by a Butterworth filter and ordered-subset expectation maximization iterative reconstruction. An ROI of 16 pixels$\times$3 slices was drawn over bilateral condylar heads. The individual condylar counts within the ROIs were calculated and the clivus bone was used as a control [14, 15], and calculation of the uptake ratio was performed by the following formula:

Condylar uptake =$$\frac{\text{c}\text{o}\text{n}\text{d}\text{y}\text{l}\text{e} \text{c}\text{o}\text{u}\text{n}\text{t}\text{s}}{\text{L}\text{e}\text{f}\text{t} \text{c}\text{o}\text{u}\text{n}\text{t}\text{s}+\text{r}\text{i}\text{g}\text{h}\text{t} \text{c}\text{o}\text{u}\text{n}\text{t}\text{s}}\times 100$$

Condylar uptake of at least 55% with a difference of at least 10% between both sides, was regarded as active unilateral condylar hyperplasia on the reference standard [8]. All ROI analyses and image interpretations were acquired by a Xeleris 3 workstation (GE Healthcare, USA).

#### Computed tomography scan reconstruction and 3D measurement

**Fig. 1:**
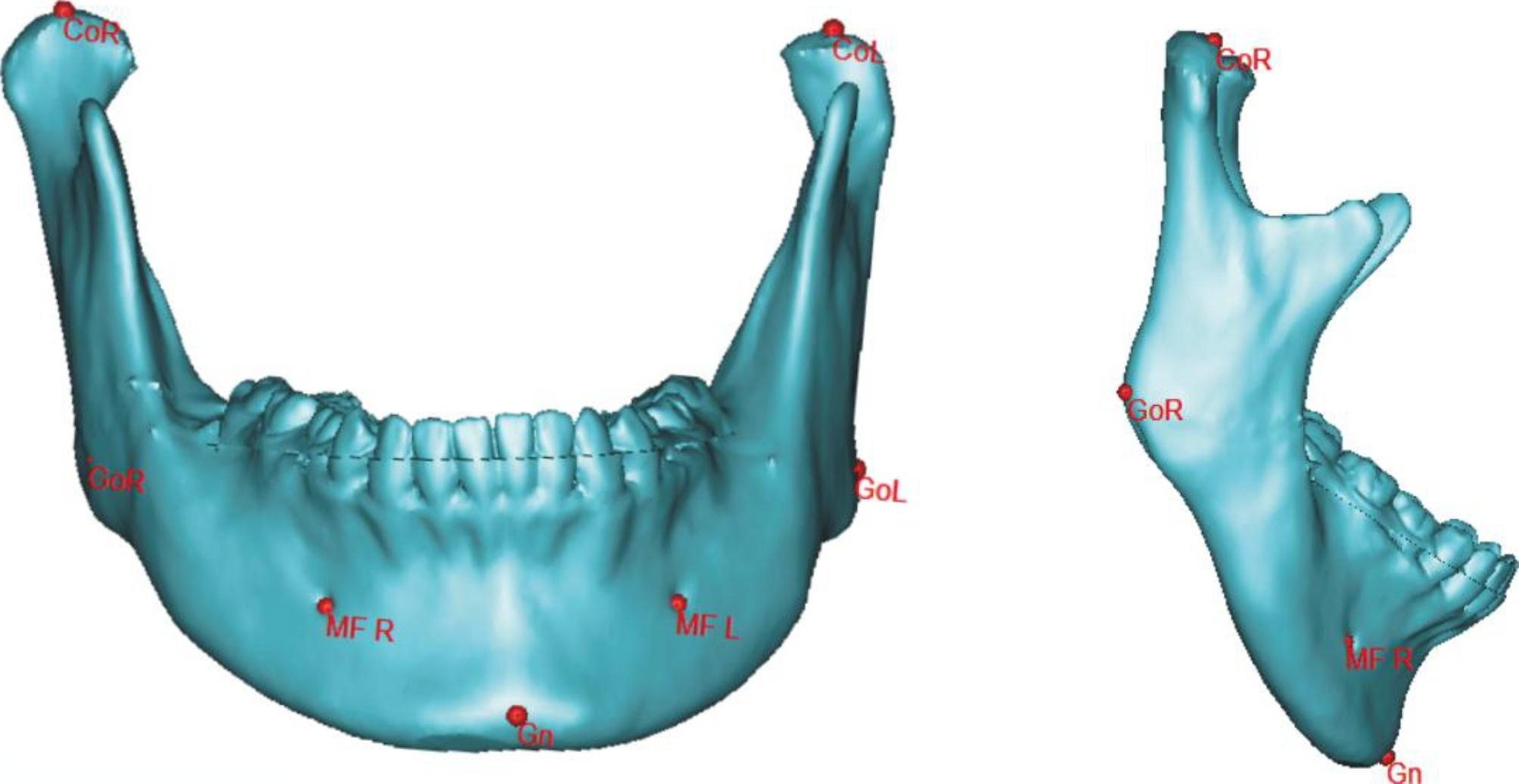
Diagram of landmarks for 3D measurements. CoR, CoL, GoR, GoL, MFR, MFL, and Gn were defined as the diagram shows

Parameters of the CT scan (GE Healthcare, USA) were 120 kV and 178 mAs. The slice thickness of the CT image was 1.25 mm, and CT scans were reconstructed and brought into 3D measurements with Proplan Software (Edition 1.4, Materialise, Leuven, Belgium) as we previously described [16]. Landmarks and measurement parameters are shown in Fig. 1; Table 1.

### Statistical analysis

Data from 3D measurements were presented as mean ± SD and the bilateral difference between two CT scans (△CT2-CT1) was analyzed with ANOVA. The sensitivity and specificity of SPECT were calculated based on the 3D measurements of CT scans, and the receiver operating characteristic (ROC) curve was drawn, with the area under the ROC curve (AUC) calculated to reflect the diagnostic capability of SPECT. Pearson’s correlation analysis was performed on the 3D measurement parameters and difference in SPECT uptake ratios to investigate whether the mandibular growth was correlated with SPECT value. To ensure the reliability of the measurement data, the 3D measurement was repeated by the same measurer at an interval of two weeks and the average value of the two measurements was taken as the final value. Intraclass correlation coefficient (ICC) was used to assess intra-investigator reliability. IBM SPSS software (New York, USA) was used for statistical analysis. A level of significance of 5% was applied. *P* < 0.05 was considered to demonstrate statistically significant differences.

**Table 1:** Definition of 3D landmarks and measurement parameters

Landmark Measurements	Definition
**CoL**	exact tip of left condyle
**CoR**	exact tip of right condyle
**GoL**	left midpoint of posterior border of mandibular angle
**GoR**	right midpoint of posterior border of mandibular angle
**MFL**	left foramina located on the anterior surface of the mandible
**MFR**	right foramina located on the anterior surface of the mandible
**Gn**	point located perpendicular on madibular symphysis midway between pogonion and menton
**Co-Gn**	anterior-posterior length of mandible
**Co-Go**	the ramus height of mandible
**Go-Gn**	the length of mandibular body
**Go-MF**	length of posterior half of mandibular body
**MF-Gn**	length of mesial half of mandibular body

Abbreviations: CoL: left condylion, CoR: right condylion, GoL: left gonion, GoR: right gonion, MFL: left mental foramen, MFR: right mental foramen, Gn: gnathion

## Results

**Fig. 2:**
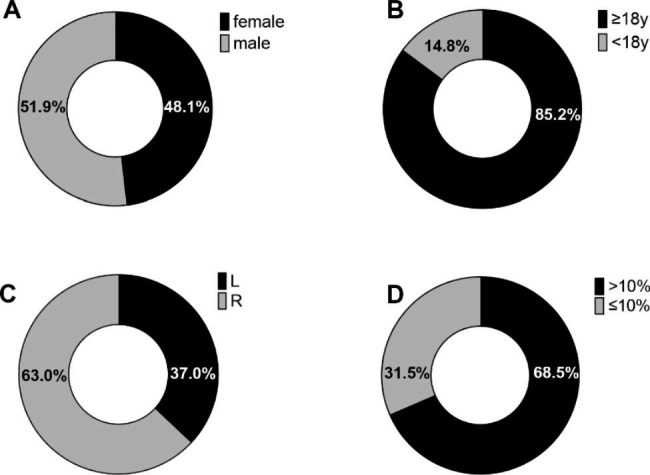
Demographic characteristics and basic diagnostic information of UCH patients. Distribution of gender (**A**), age (**B**), affected side (L means left side and R means right side) (**C**), and relative condylar uptake ratios (**D**)

Data of fifty-four patients (28 male and 26 female) diagnosed with UCH were analyzed retrospectively in this study, with an average age of 19.87 ± 2.76 years old. Demographic characteristics and basic diagnostic information are shown in Fig. 2. The left condyle was affected in 20 cases, while the right condyle was affected in 34 cases. The difference in relative condylar uptake ratios was recorded, ranging from 1 to 28%, with a median uptake ratio of 13.11 ± 7.66%. No significant difference was observed between male and female patients or between patients over 18 years old and under 18 years old (Tables 2 and 3).

**Table 2:** SPECT value (difference in relative condylar uptake ratios) according to gender

SPECT Value				
	Mean	SD	Minimum	Maximum
Male	14.5%	11.62%	1%	35%
Female	11.62%	14.5%	1%	23%
*p* = 0.1689				

Abbreviation: SD, standard deviation

**Table 3:** SPECT value (difference in relative condylar uptake ratios) according to age

		SPECT Value			
	Mean		SD	Minimum	Maximum
<18y	12.25%		4.097%	6%	18%
≥ 18y	13.26%		8.144%	1%	35%
*p* = 0.7340					

Abbreviation: SD, standard deviation

**Fig. 3:**
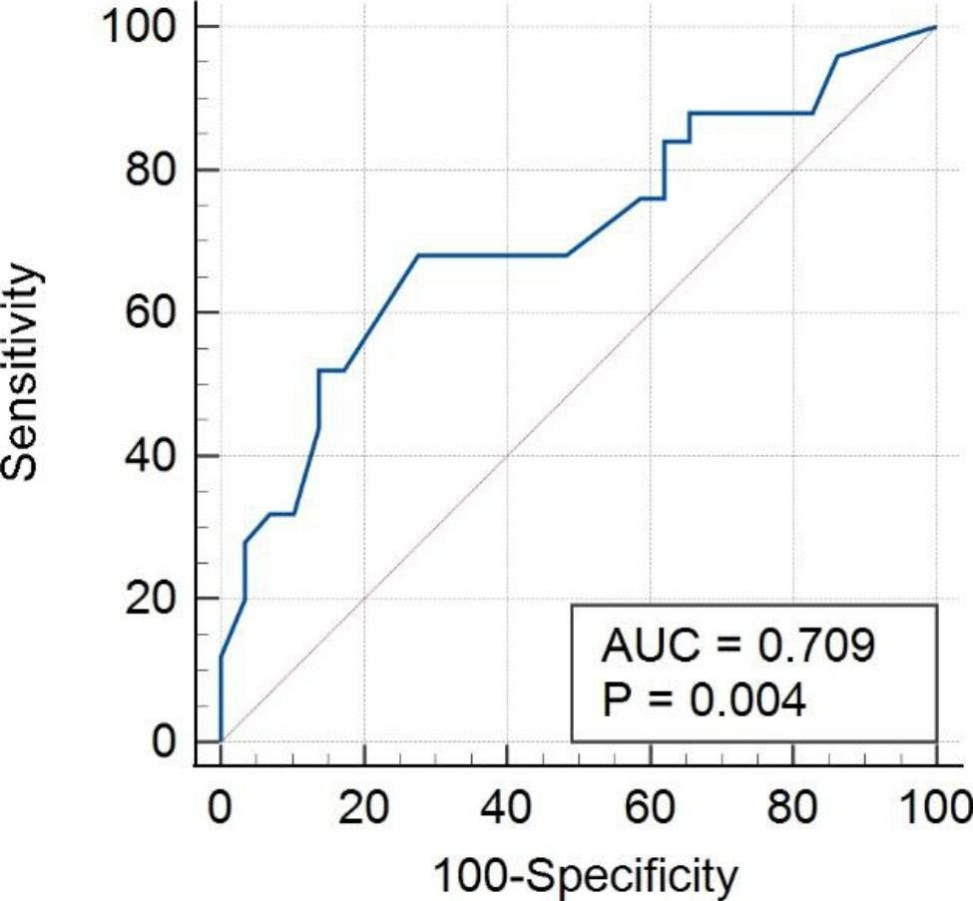
Receiver operating characteristic (ROC) curve for SPECT AUC, 0.79; 95% CI: 0.569 − 0.825. Youden index J: 0.4041. AUC, the area under the curve; CI, confidence interval

The sensitivity of SPECT was 68.00%, and the specificity was 72.41% (Table 4). The ROC curve in Fig. 3 illustrated that SPECT could be used to determine whether the condyle is active or inactive based on the difference in relative uptake ratios, with 13% as the optimal cut-off value (Table 4). The AUC was 0.709, and the 95% confidence interval (95% CI) was 0.569 to 0.835. In the case where the difference in relative condylar uptake ratios was greater than 23%, the specificity was 100%, which indicated that all the patients were in an active condylar growth state, and this group of UCH patients was defined as population S (Table 5).

**Table 4:** Comparison of SPECT and clinical/ radiological assessments

	Clinical and radiological assessment		
SPECT assessment (13%)	Active	Inactive	Total
Active	17	8	25
Inactive	8	21	29
Total	25	29	54
True positive (sensitivity)	17(68.00%)		
True negative (specificity)	21(72.41%)		

**Table 5:** Criterion values and coordinates of the ROC curve

Criterion	Sensitivity	95% CI	Specificity	95% CI		
> 8%	84.00	0.639–0.955	37.93	0.207–0.577		
> 10%	76.00	0.549–0.906	37.93	0.207–0.577		
> 11%	76.00	0.549–0.906	41.38	0.235–0.611		
> 12%	68.00	0.465–0.851	51.72	0.325–0.706		
> 13%	68.00	0.465–0.851	72.41	0.528–0.873		
> 23%	12.00	0.025–0.312	100	0.881–1		

Abbreviation: CI, confidence interval

**Fig. 4:**
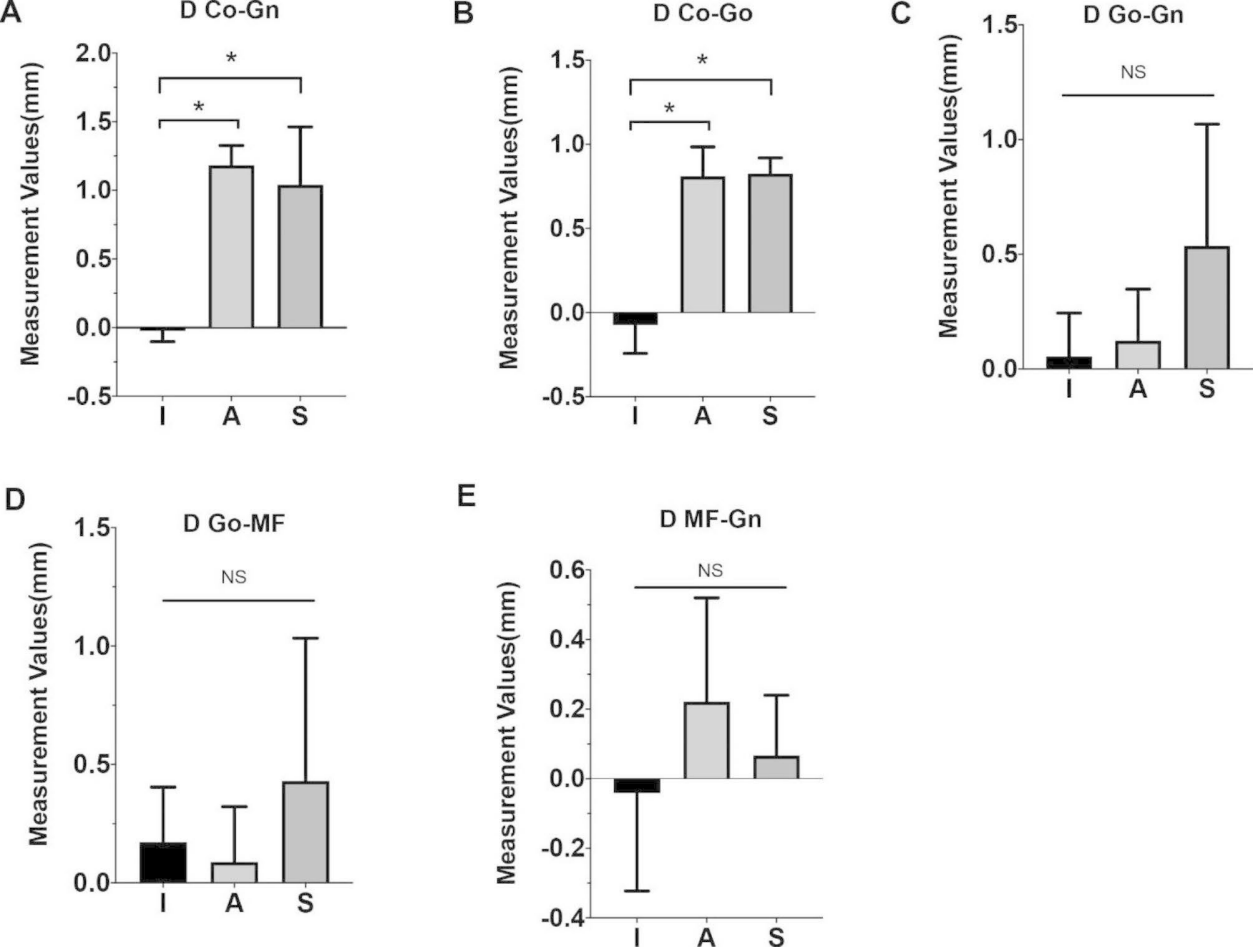
Changes in 3D measurement of the mandible in population I, A, and S. Changes in CT scan of the follow-up visit (CT2) compared with initial CT scan (CT1) of Co-Gn, Co-Go, Go-Gn, Go-MF, and MF-Gn in population I, A and S. Data were calculated by comparing changes in bilateral difference (affected side vs. unaffected side). *p < 0.05, NS: no significant difference

The average intra-operator ICC for 3D-measurement of the CT scan was 0.956. The results in Fig. 4 demonstrated that there was a significant increase in Co-Gn (△CT2-CT1) in population A and S when compared to population I (△=1.208 ± 0.159 mm, *p*<0.05; △=1.065 ± 0.353 mm, *p*<0.05), indicating unbalanced growth unilaterally in the diagonal direction of the mandible. Meanwhile, as for Co-Go (△CT2-CT1), which indicates growth in the ramus height of the mandible, the increase was also significantly greater in population A compared to population I (△=0.884 ± 0.229 mm, *p*<0.05), as well as in population S compared to population I (△=0.901 ± 0.320 mm, *p*<0.05). Furthermore, changes in parameters related to mandibular body length, including Go-Gn, Go-MF, and MF-Gn, were observed in population I, A, and S. However, no significant difference was detected. Notably, with all P values greater than 0.05, Pearson’s correlation analysis revealed no correlation between 3D measurement parameters and the difference in relative condylar uptake ratios, which indicated that SPECT and radiological 3D measurement may both be needed in the evaluation of the UCH activeness in clinical practices (Table 6).

**Table 6:** Pearson’s Correlation Analysis of the Difference in Condylar Uptake Ratios and 3D Measurement Parameters

Assessment Parameter	D Co-Gn	D Co-Go	D Go-Gn	D MF-Gn	D Go-MF
Pearson correlation coefficient	0.253	0.234	-0.020	-0.213	0.064
*P* value	0.065	0.088	0.886	0.121	0.644

## Discussion

It’s critical to distinguish the mandibular condyle’s active and inactive stages in managing UCH. Various methods have been used to evaluate the growth of the mandible, including clinical examination, dental casts, X-rays, and cephalometric analysis [12, 17–19]. Nevertheless, factors like body posture and mannerism can conceal the growth imbalance, which might lead to incorrect treatment decisions [20]. Hence, alternative diagnostic procedures such as 99mTc-MDP bone scintigraphy and SPECT should be used in conjunction with clinical examination [21–24]. In recent decades, evaluation of condylar growth activeness of UCH with SPECT is increasingly recommended, with an optimal diagnostic threshold of 10% difference in relative uptake ratios [25]. Hodder et al. [17] used SPECT as an aid to diagnosis and confirmed that an uptake ratio of more than 10% was considered as active condylar hyperplasia, together with appropriate clinical and plain radiographic features. On the other hand, Kajan et al. [12] determined that the variation in growth activity in normal mandibular condyles was less than 6.2%. Similiarly, Fernandes et al. [1] concluded that the variation in condylar uptake was less than 5% in 37 out of 44 TMJ ‘normal’ patients, and in none was it 9% or more. However, the condylar uptake difference greater than 10% was not always acknowledged as the standard reference for predicting active condyle growth [12–14]. We assume the discrepancies in the diagnostic threshold of relative uptake ratios among studies might be due to different sample sizes, diverse assessment methods, and possible racial differences. Crucially, the difference in relative uptake ratios of UCH in the Chinese population has not been identified before. Based on the ROC curve drawn by SPECT assessment, this study determined that 13% is the optimal cut-off value for evaluating condyle activity in Chinese UCH patients for the first time. In ROC curve analysis, we discovered that SPECT had a sensitivity of 68.00% and a specificity of 72.41%, yielding an AUC of 0.709, indicating that SPECT is a valuable tool in measuring condylar growth activity. We also discovered that when the relative condylar uptake ratios differed by more than 23%, all the patients were defined as active, suggesting a ‘definite’ active condition.

Our results could explain why some studies concluded that SPECT was not a suitable diagnostic tool for evaluating condylar growth, as the criterion was set at 10% or the assessment was conducted with an improper method or based on a 2D imaging approach [14, 26]. Xiao et al. [26] adopted 3D measurement results as the gold standard, defining a difference of more than 5% in Co-Go, Go-Pg, Co-B, or condylar volume between two visits as active in assessing condylar growth status, and thus concluded that SPECT had poor sensitivity and specificity. However, this gold standard may have disproportionately increased the number of cases diagnosed as active UCH due to the large uncertainty and manual variance in the measurement of condylar volume. As Gateno et al. [27] observed that UCH could expand a hemimandible with or without considerable condylar enlargement and there was no correlation between condylar enlargement and overgrowth direction. The specificity of SPECT would inevitably diminish if the number of ‘active’ instances increased excessively.

UCH can occur at any age [28]. In our study, the mean age of the patients was 19.87 ± 2.76 years old, ranging from 16 to 29 years old, with the right condyle being more frequently affected (63%). Interestingly, Martin-Granizo et al. [29] reported the similar proportion. Meanwhile, some researchers revealed the same tendency regarding the side of laterognathia [30], while others suggested that difference in the affected side was not statistically significant [10, 31]. Female patients accounted for 26 (48.1%) and male patients accounted for 28 (51.9%) herein, which appears to counter earlier findings indicating UCH mostly affects women [27]. The discrepancy might be explained by the fact that patients in our research only represent a biased sample with active hyperplasia condyles and follow-up visits, also suggesting that male patients were more likely to require follow-up visits. Females were found to present with UCH at a younger age, and as a consequence, they ceased their growth activity earlier, whereas male patients were found to present with UCH later, and as a result, they ceased their growth activity later [14]. However, the distribution pattern of SPECT values among UCH patients has not been studied. Our study first investigated the differences in SPECT uptake ratios by age and sex in a Chinese population of UCH patients and observed no significant differences between male and female patients, or between patients over 18 years old and under 18 years old.

In 1986, Obwegeser first proposed that UCH is consistent with two independent conditions: hemimandibular hyperplasia and hemimandibular elongation. In hemimandibular hyperplasia, the volume of half the mandible increases, since the condyle expands, the neck widens and lengthens, and the ramus and body heighten. In hemimandibular elongation, one side of the mandible lengthens in a horizontal plane, shifting the chin to the unaffected side, as the condyle remains unchanged or slightly expands. The neck can lengthen, however, this is not always the case [3]. The following three assumptions guide the above classification: First, UCH overgrowth is bimodal, developing vertical or horizontal in some cases, and diagonally in others; Second, hyperplasia can be either global or linear, with global hyperplasia enlarging the condyle and the rest of the hemimandible, and linear hyperplasia lengthening one side of the jaw while maintaining the condyle’s previous size; Third, the direction of overgrowth is determined by the type of hyperplasia, as vertical growth is produced by global hyperplasia, whereas horizontal growth is produced by linear hyperplasia [32]. Herein, we found a significantly unbalanced growth unilaterally in the diagonal direction of the mandible and considerably greater growth in the ramus height of the mandible in population A compared to population I. Furthermore, no significant difference was detected in parameters related to mandibular body length in either group. Similar findings were found in the recent scientific literature on mandibular structure measurement of patients with UCH. Gateno et al. [27] investigated that the growth vectors in UCH are unimodally distributed, with the majority expanding diagonally. Lopez et al. [33] indicated that patients with UCH displayed larger condylar length and volume on the affected side. Evangelista et al. [34] concluded that the mandibular body length was more correlated to mandibular rotation than condylar differences, which might explain our study’s undifferentiated mandibular body length.

Furthermore, according to our study, the relative condylar uptake ratio was not directly related to mandibular growth. Contrary to CT scans and X-rays that require greater changes in bone density for the anatomical alteration to be evident, bone scans are sensitive examinations of the entire skeleton dependent on blood flow and absorption into hydroxyapatite crystals; as a result, SPECT could detect early-stage alterations without observable anatomical changes in radiographs or CT scans [35, 36]. This could be the explanation why there is no statistically significant association between positive SPECT results and changes in CT scans. Additionally, in cases of active condylar hyperplasia, alterations can occur in the condylar volume with anatomical modification of the joint cavity without necessarily projecting the entire modification in a sagittal or transverse direction at the mandibular level. As Lopez et al. [33] reported a positive correlation between the increased dimensions of the articular eminence and the more posterior position of the glenoid fossa in the affected side. According to their latest research which is contrary to our results, Lopez et al. [36] concluded that there is a significant correlation between the magnitude of mandibular deviation quantified on CT and metabolic findings obtained by SPECT in patients with UCH. Further studies on the correlation between SPECT value and mandibular structure should be carried out in the subsequent research.

Finally, the following are the main limitations of this study: (1) We acknowledge that the study’s principal limitation was the retrospective nature of the research, which might lead to biases. (2) The study was conducted at a single center with a limited sample of the population. As a result, extending the findings from this study to a different ethnic group may not be entirely accurate. For further evaluation, multicenter and larger-sample studies should be conducted. (3) There was still an average inaccuracy in the repeated 3D measurements. (4) With larger samples, studies of growth prediction in UCH patients using cluster and discriminant function analysis should be further performed.

## Conclusion

In conclusion, the sensitivity and specificity of SPECT for identifying active condylar growth were 68.00% and 72.41%, respectively, demonstrating good diagnostic performance in UCH with the cut-off value of 13%. For Chinese UCH patients with an active growing condyle, the mandible of the vast majority grows diagonally and vertically, while the relative condylar uptake ratio was not directly related to mandibular growth.

## Data Availability

Datasets used and/or analyzed during the current study are available from the corresponding author on reasonable request.
